# Morphology and Transcriptome Analysis of Nosema bombycis Sporoplasm and Insights into the Initial Infection of Microsporidia

**DOI:** 10.1128/mSphere.00958-19

**Published:** 2020-02-12

**Authors:** Qiang He, Jian Luo, Jin-Zhi Xu, Chun-xia Wang, Xian-zhi Meng, Guo-Qing Pan, Tian Li, Ze-Yang Zhou

**Affiliations:** a State Key Laboratory of Silkworm Genome Biology, Southwest University, Chongqing, China; b Chongqing Key Laboratory of Microsporidia Infection and Control, Southwest University, Chongqing, China; c College of Life Science, Chongqing Normal University, Chongqing, China; University of Georgia

**Keywords:** sporoplasm, microsporidia, *Nosema bombycis*, morphology, infection mechanism, transcriptome, metabolic pathway

## Abstract

Microsporidia are obligate intracellular parasites that infect a wide variety of host organisms, including humans. The sporoplasm is the initial stage of microsporidian infection and proliferation, but its morphological and molecular characteristics are poorly understood. In this study, the sporoplasm of Nosema bombycis was successfully isolated and characterized after the induction of spore germination *in vitro*. The sporoplasm was spherical, 3.64 ± 0.41 μm in diameter, had the typical two nuclei, and was nonrefractive. Scanning and transmission electron microscopy analyses revealed that the sporoplasm was surrounded by a single membrane, and the cytoplasm was usually filled with relatively homogeneous granules, possibly ribosomes, and contained a vesicular structure comprising a concentric ring and coiled tubules. Propidium iodide staining revealed that the sporoplasm membrane showed stronger membrane permeability than did the cell plasma membrane. Transmission electron microscopy (TEM) revealed that the sporoplasm can gain entry to the host cell by phagocytosis. Transcriptome analysis of mature spores and sporoplasms showed that 541 significantly differentially expressed genes were screened (adjusted *P* value [*P*_adj_] < 0.05), of which 302 genes were upregulated and 239 genes were downregulated in the sporoplasm. The majority of the genes involved in trehalose synthesis metabolism, glycolysis, and the pentose phosphate pathway were downregulated, whereas 10 transporter genes were upregulated, suggesting that the sporoplasm may inhibit its own carbon metabolic activity and obtain the substances required for proliferation through transporter proteins. This study represents the first comprehensive and in-depth investigation of the sporoplasm at the morphological and molecular levels and provides novel insights into the biology of microsporidia and their infection mechanism.

**IMPORTANCE** Once awoken from dormancy, the cellular matter of microsporidia is delivered directly into the host cell cytoplasm through the polar tube. This means that the microsporidia are difficult to study biologically in their active state without a contaminating signal from the host cell. Sporoplasm is a cell type of microsporidia *in vitro*, but relatively little attention has been paid to the sporoplasm in the past 150 years due to a lack of an effective separation method. Nosema bombycis, the first reported microsporidium, is a type of obligate intracellular parasite that infects silkworms and can be induced to germinate in alkaline solution *in vitro*. We successfully separated the *N. bombycis* sporoplasm *in vitro*, and the morphological and structural characteristics were investigated. These results provide important insight into the biology and pathogenesis of microsporidia and potentially provide a possible strategy for genetic manipulation of microsporidia targeting the sporoplasm.

## Abstract (precis)

Once awoken from dormancy, the cellular matter of microsporidia is delivered directly into the host cell cytoplasm through the polar tube. This means that the microsporidia are difficult to study biologically in their active state without a contaminating signal from the host cell. Sporoplasm is a cell type of microsporidia *in vitro*, but relatively little attention has been paid to the sporoplasm in the past 150 years due to a lack of an effective separation method. Nosema bombycis, the first reported microsporidium, is a type of obligate intracellular parasite that infects silkworms and can be induced to germinate in alkaline solution *in vitro*. We successfully separated the *N. bombycis* sporoplasm *in vitro*, and the morphological and structural characteristics were investigated. These results provide important insight into the biology and pathogenesis of microsporidia and potentially provide a possible strategy for genetic manipulation of microsporidia targeting the sporoplasm.

## INTRODUCTION

Microsporidia are a type of obligate intracellular eukaryotic parasite hosted by a wide diversity of animals, including humans and commercially important insects (1, 2). At present, more than 1,500 species of microsporidia have been identified (3, 4). In recent decades, microsporidia have attracted increasing attention because they represent a connection between pathogen contamination and human food chains (5). Microsporidia can only complete their life cycle inside a host cell, owing to a reduction in metabolic capabilities and expansion of transporter gene families (6–9).

Microsporidia can survive in the external environment as highly resistant spores with a thick two-layered wall, and they contain a highly specialized invasion apparatus called the polar tube used to release sporoplasm (3, 10–12). After the successful transfer of sporoplasm into the host cell cytoplasm, the infective sporoplasm enters the proliferative stage and completes the life cycle (11, 13). Therefore, the sporoplasm is critical for the infection and proliferation of microsporidia, and thus, an investigation of the sporoplasm will provide valuable information for understanding the biological characteristics of microsporidia (14, 15). The sporoplasms are spherical-to-ovoid cells with a typical plasma membrane, approximately 1.5 to 2.0 μm in diameter (16, 17). The appearance of the sporoplasm varies among different organisms and may vary within the same organism in different environments. The plasma membrane of the sporoplasm ejected from the polar tube may be derived from the polaroplast (18). Many organelles are detectable in the sporoplasm by transmission electron microscopy (TEM) observation, such as polyribosomic clusters, Golgi-related structures, and the endoplasmic reticulum (15, 19, 20). A sporoplasm in the process of being phagocytized into the host cell by pseudopods can be observed. The sporoplasm of Spraguea lophii can survive for about 24 h in Medium 199 (Sigma-Aldrich) enriched with ATP *in vitro* (21). However, little attention has previously been paid to the sporoplasm, which is difficult to observe, and currently, the spores of only a small number of species can be activated *in vitro* using a germination solution (15, 16, 20, 22). Furthermore, sporoplasms are difficult to identify and separate in the host cytoplasm on account of their small size, lack of chitin, and absence of a fixed form (16, 23).

Germination of microsporidia is the means of releasing sporoplasms, which involves a series of biologically complex changes (24). To elucidate the molecular basis of the germination process, transcriptome sequencing and quantitative proteomic analysis of ungerminated and germinated spores of *N. bombycis* have been investigated (25, 26). Several genes associated with germination were identified, especially genes involved in protein dephosphorylation (25), and a number of important changes in metabolic pathways were detected during the germination process, such as glycolysis, the pentose phosphate pathway, and purine and pyrimidine metabolism (26). After the infective sporoplasm enters the host cytoplasm, it develops into meronts and undergoes proliferation, which demands much energy (7, 27). Therefore, microsporidia steal ATP from the host cell by means of an ATP/ADP carrier protein and switch off their own energy metabolism during intracellular development (28–30). For example, phosphoglycerate kinase 3 (PGK-3), which catalyzes the ATP-forming reaction of glycolysis, was revealed by immunolabeling to accumulate specifically in the spore stage and not the meront stage of Trachipleistophora hominis (8). Given that the sporoplasm is the crucial stage of the microsporidial life cycle for progression from dormancy to proliferation, an in-depth understanding of sporoplasm biology will provide novel insights into the invasion and propagation processes. However, to date, this stage remains poorly understood at the molecular level because of the difficulties in isolating sporoplasms.

In this study, we provide the first example of using germination *in vitro* to isolate the *N. bombycis* sporoplasm and analyze the morphological and structural characteristics of the sporoplasm. In addition, the transcriptomes of the mature spore (MS) and sporoplasm (SP) of *N. bombycis* were analyzed. The results contribute to an improved understanding of the sporoplasm at the morphological and molecular levels.

## RESULTS

### Isolation and size of sporoplasm.

**FIG 1:**
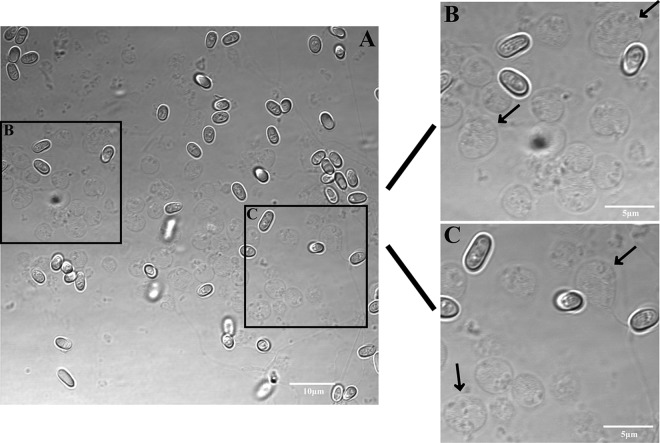
Light microscopic observation of *N. bombycis* sporoplasm morphology. (A) Sporoplasm extruded through the polar tube. (B and C) Enlargement of the black rectangle in panel A highlighting the sporoplasm (black arrows).

**FIG 2:**
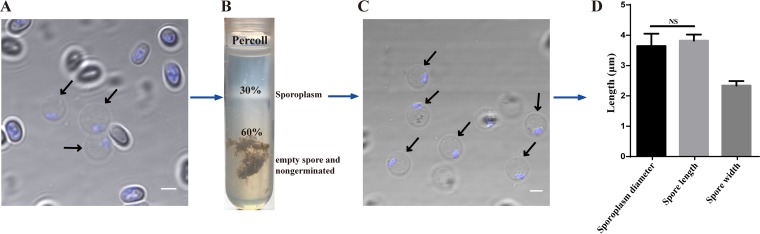
Isolation and purification of the sporoplasm. (A) Sporoplasm stained with Hoechst 33258 (nuclei, blue). Black arrow indicates the sporoplasm. Scale bar = 2 μm. (B) Sporoplasm purified from incubated mixture using 30% and 60% Percoll gradient centrifugation. Sporoplasm was predominantly isolated in 30% Percoll. (C) Sporoplasm was verified in 30% Percoll. Red arrow indicates the sporoplasm. Scale bar = 2 μm. (D) Comparison of the average diameters of the sporoplasm and mature spore. Vertical bars represent the mean ± standard error (SE) (*n *= 80).

To isolate sporoplasms, mature spores of *N. bombycis* were induced to germinate in alkaline solution, and the germination solution was replaced with an isotonic cell medium to avoid cell rupture. Light microscopic observation revealed that the sporoplasm was pear shaped immediately after extrusion through the polar tube and was nonrefractive (Fig. 1). The sporoplasm diameter ranged from 2.7 to 5.2 μm. After several minutes, all sporoplasms were spherical and averaged 3.64 ± 0.41 μm in diameter (*n *= 80) with the typical two nuclei located close to the cell membrane (Fig. 2A and D). At this stage, sporoplasms were purified by means of Percoll gradient centrifugation and were mainly distributed in the 30% Percoll layer (Fig. 2B and C).

### Sporoplasm ultrastructure.

**FIG 3:**
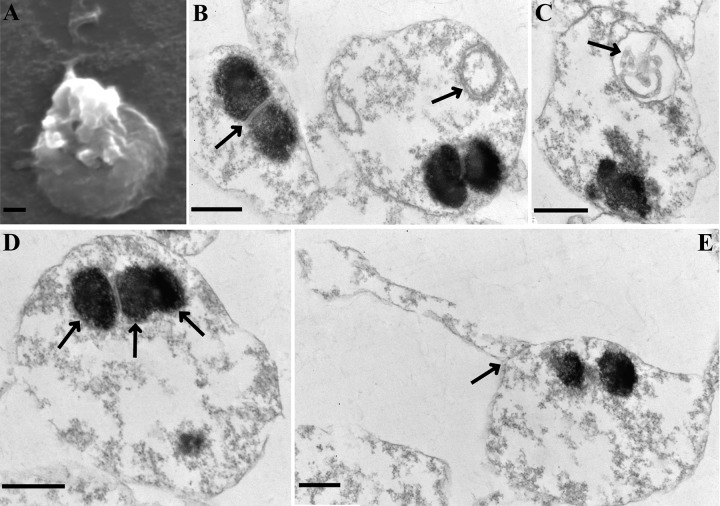
Ultrastructure of sporoplasm. (A) Scanning electron micrograph of a sporoplasm. (B to E) Transmission electron micrographs of sporoplasm. (B) Connection between two nuclei (indicated by black arrows). A vesicular structure consisting of a concentric ring and/or containing coiled tubules was observed in the cytoplasm (indicated by black arrows in panels B and C). A sporoplasm containing three nuclei was observed (indicated by black arrows in panel D). (E) After germination, the sporoplasm remained attached to a tubular structure surrounded by a membrane. Scale bar = 500 nm.

Scanning electron microscopy (SEM) revealed that sporoplasms were spherical and wrinkled and remained attached to the polar tube after germination (Fig. 3A). Observation by TEM showed that the sporoplasm was surrounded by a smooth single membrane and contained diplokaryotic nuclei, which were encompassed by a typical plasma membrane (Fig. 3B). The surface of the sporoplasm was connected to the residual polar tube, and a distinct interval was observed between the polar tube and the sporoplasm, which may facilitate the shedding of the polar tube (Fig. 3E). Three nuclei were observed in a sporoplasm (Fig. 3D). The mean length of the nuclei of the mature spores (0.78 ± 0.07 μm) and sporoplasms (0.59 ± 0.05 μm) were measured by TEM (*n *= 50) (see Fig. S1 in the supplemental material). These results suggested that the sporoplasm appeared to be in a flexible state, being conducive extruded from the polar tube. The cytoplasm of the sporoplasm was usually filled with relatively homogeneous granules, possibly ribosomes, and contained a vesicular structure comprising a concentric ring and coiled tubules (Fig. 3B and C). Other organelles were not detected in the sporoplasm.

10.1128/mSphere.00958-19.1 FIG S1 Sporoplasm nucleus is compacted after germination. (A and B) Transmission electron micrograph of sporoplasm and mature spore nuclei. Scale bar = 500 nm. (C) Comparison of average nucleus lengths in the sporoplasm and mature spores by TEM. Statistically significant diﬀerences are indicated with asterisks (**, *P < *0.01). Download FIG S1, TIF file, 2.5 MB. Copyright © 2020 He et al. 2020 He et al. This content is distributed under the terms of the Creative Commons Attribution 4.0 International license.

### Characterization of sporoplasm membrane.

**FIG 4:**
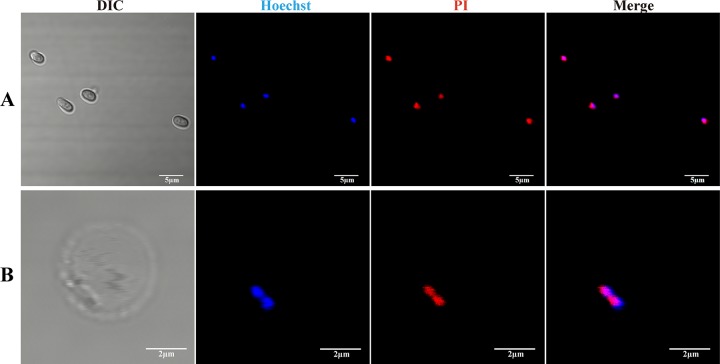
Fluorescent labeling of sporoplasm membrane. The plasma membrane was stained with DiI (red) and wheat germ agglutinin (green); the nucleus was stained with 4′,6-diamidino-2-phenylindole (DAPI) (blue). Scale bar = 2 μm. DIC, differential interference contrast.

**FIG 5:**
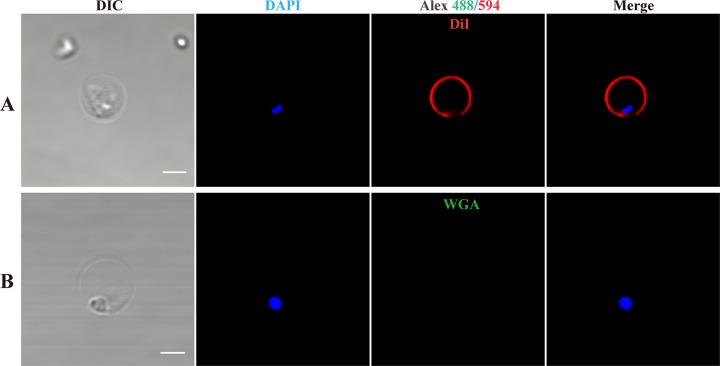
Propidium iodide staining of sporoplasm and mature spore. (A and B) The nucleus was labeled by Hoechst 33258 (blue) and PI (red) in the mature spore (A) and sporoplasm (B).

The sporoplasm membrane was stained with DiI, which is a type of lipophilic membrane dye, but not by wheat germ agglutinin (WGA), which binds to sialic acid and *N*-acetylglucosaminyl residues (Fig. 4). The mature spore was stained by WGA and the plasma membrane of the host cell was stained by DiI and WGA (Fig. S2). These results demonstrated that the sporoplasm membrane lacked lectin-binding sites. The sporoplasm and mature spore were stained with propidium iodide (PI), which can label the nucleus as an indicator of plasma membrane permeability. Both the mature spore and sporoplasm nuclei exhibited red fluorescence after staining with PI and blue fluorescence after staining with Hoechst 33258, which indicated that the sporoplasm membrane was strongly permeable (Fig. 5).

10.1128/mSphere.00958-19.2 FIG S2 (A) Mature spore stained with WGA. (B and C) Plasma membrane of SF9 cell stained with wheat germ agglutinin (green) and DiI (red). Download FIG S2, TIF file, 2.9 MB. Copyright © 2020 He et al. 2020 He et al. This content is distributed under the terms of the Creative Commons Attribution 4.0 International license.

### Sporoplasm phagocytosis by host cells.

**FIG 6:**
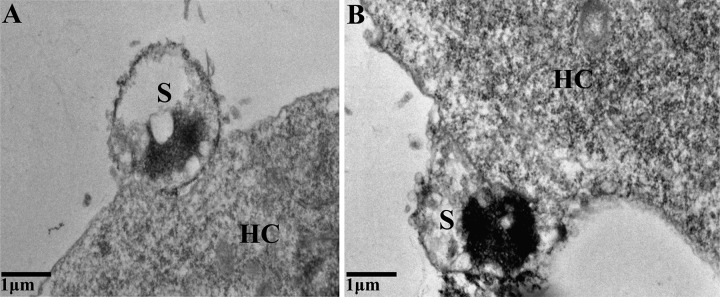
Transmission electron micrograph of sporoplasm phagocytized by the host cell. The sporoplasm is bound to the SF9 cell membrane by pseudopod-like protrusions. S, sporoplasm; HC, host cell.

**FIG 7:**
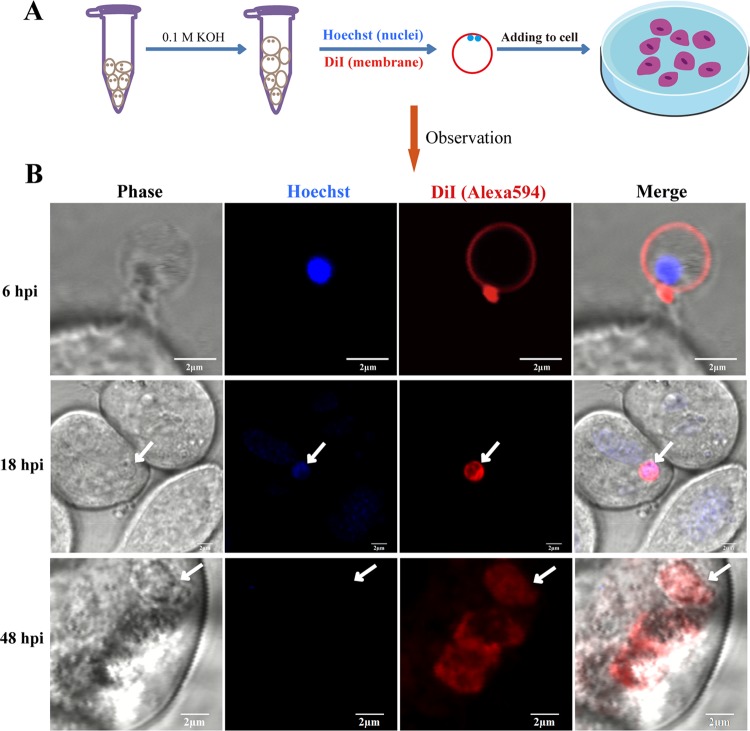
Continuous fluorescent observation of sporoplasm that infected the host cells. (A) Diagram of the experimental process. (B) The sporoplasm was stained with Hoechst 33258 (blue) and DiI (red) and placed in a petri dish containing the SF9 cell line at 6, 18, and 48 h postinfection (hpi). The white arrow indicates a sporoplasm emitting red fluorescence.

Sporoplasms were added to culture dishes and using TEM were observed to attach to cells by a membrane-derived protrusion from the host cell surface (Fig. 6). However, phagocytosis of the sporoplasm into a host cell was not observed. A fluorescence assay was used to visualize the process of host cell infection by the sporoplasm. The sporoplasm adhered to the surface of the host cell and was enveloped by protruding projections from the host cell at 6 h postinfection (hpi) (Fig. 7). After 18 hpi, the fluorescence-labeled sporoplasm had invaded the host cell, thus confirming that the sporoplasm was eventually phagocytized into the host cell. At 48 hpi, early piriform spores that exhibited red fluorescence were observed in the host cells. At this stage, the host cells were relatively healthy in appearance and remained adherent to the substrate, the plasma membranes remained intact, and no visible evidence of apoptosis was observed.

### Sequencing analysis of MS and SP transcriptomes.

RNA sequencing (RNA-seq) was used to investigate gene expression in the MS and SP of *N. bombycis* with three biological repeats (Fig. S3). After the sequencing and filtration, 146,865,590 and 135,344,316 high-quality reads (total numbers from three biological replicates) were obtained for the MS and SP groups (Table 1). The high-quality reads were mapped to the reference genome of *N. bombycis*; the matching rates for the MS and SP reads were 82.54% and 83.18%, respectively. Subsequently, we obtained evidence for the expression of 4,010 genes (89.9%) and 276 novel genes compared with the 4,460 annotated genes in the *N. bombycis* reference genome (Table S2).

**TABLE 1:** Summary of RNA sequencing of the sporoplasm (SP) and mature spore (MP) transcriptomes of *N. bombycis*^*a*^

Characteristic	SP	MS
No. of raw reads	41,315,096, 42,567,484, 53,699,680	53,097,578, 47,806,468, 48,603,210
No. of high-quality reads	40,573,148, 41,988,206, 52,782,962	52,216,732, 47,072,200, 47,576,658
Total length of clean reads (Gb)	6.09, 6.3, 7.92	7.83, 7.06, 7.14
Total no. (%) of mapped reads	33,618,253 (82.86), 34,923,587 (83.17), 44,082,015 (83.52)	43,416,833 (83.15), 38,637,857 (82.08), 39,194,217 (82.38)

a Data are given as the results from the three replicates performed.

10.1128/mSphere.00958-19.3 FIG S3 Morphology and total RNA analysis of *N. bombycis* mature spore and sporoplasm. (A) Isolation of sporoplasm. (B) Purification of mature spore. (C) The detection of total RNA. M, marker of DNA; lanes 1 to 3, three replications of sporoplasm; lanes 4 to 6, three replications of mature spore. Download FIG S3, TIF file, 2.0 MB. Copyright © 2020 He et al. 2020 He et al. This content is distributed under the terms of the Creative Commons Attribution 4.0 International license.

### Differentially expressed gene analysis.

**FIG 8:**
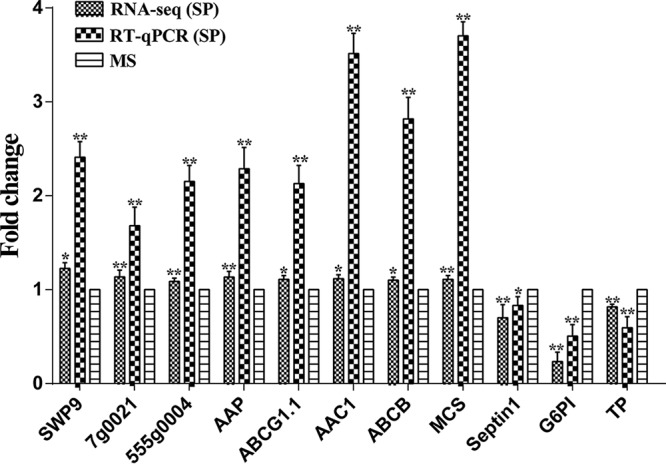
Relative expression levels of 11 randomly selected genes measured by RT-qPCR and RNA-seq. SWP9, spore wall protein 9; AAP, amino acid permease; ABCG1.1, ATP-binding cassette transporter G member 1.1; AAC1, ADP/ATP transporter protein; ABCB, ATP-binding cassette transporter B; MscS, small-conductance mechanosensitive channel protein; G6PI, glucose-6-phosphate isomerase; TP, trehalose-phosphatase. Transcription levels were calculated using the 2^−ΔΔ^*^CT^* method with three replicates. *NbSSU* was used as the reference gene. Data are presented as the mean ± standard deviation (SD) of the results from three independent biological replications. The fold change of each gene was determined by three independent quantitative PCR amplifications of RNA extracted independently. Statistically significant differences are indicated with asterisks (*, *P* < 0.05; **, *P* < 0.01).

By calculating the fragments per kilobase per million (FPKM) values of the expressed genes in the SP and MS groups, a total of 541 significantly differentially expressed genes (DEGs) were screened (|log_2_[fold change]|, >0; *P*_adj_ < 0.05), of which 302 genes were upregulated and 239 genes were downregulated in the sporoplasm (Table S3). A total of 361 genes were annotated using the nonredundant (NR) protein database. To verify the DEGs identified by RNA-seq, real-time quantitative PCR (RT-qPCR) was utilized to detect the differential expression levels of 11 randomly selected genes (Fig. 8). The expression patterns of all selected genes measured by RNA-seq and RT-qPCR were identical, thus showing consistency in the results of the DEG analysis.

### Functional enrichment of DEGs.

To explore the biological functions of the DEGs, Gene Ontology (GO) analysis was performed based on GO annotation terms. Enriched GO terms were classified to the biological process (BP), cellular component (CC), and molecular function (MF) classes. The DEGs were enriched in 327 GO terms, and the 10 most highly significant GO terms were selected for display (Table S3 and Fig. S4). The DEGs were involved in the amide biosynthesis process and organonitrogen compound biosynthesis process in the BP class, with ribosomes in the CC class, and with structural constituents of ribosomes and structural molecule activity in the MF class (Fig. S4).

10.1128/mSphere.00958-19.4 FIG S4 GO function enrichment analysis of differentially expressed genes. BP, biological process; CC, cellular component; MF, molecular function. Download FIG S4, TIF file, 0.7 MB. Copyright © 2020 He et al. 2020 He et al. This content is distributed under the terms of the Creative Commons Attribution 4.0 International license.

To identify the associated biological pathways activated during development from the mature spore to the sporoplasm, all DEGs were searched against pathway annotations in the Kyoto Encyclopedia of Genes and Genomes (KEGG) database. The DEGs were enriched in 29 KEGG pathways, including the ribosome and pentose phosphate pathways, carbon metabolism, aminoacyl-tRNA biosynthesis, and amino sugar and nucleotide sugar metabolism (Table S4 and Fig. S5). Pathways that participate in ubiquitin-mediated proteolysis and purine metabolism were also detected in the transcriptome.

10.1128/mSphere.00958-19.5 FIG S5 KEGG pathway enrichment analysis of differentially expressed genes. Download FIG S5, TIF file, 0.5 MB. Copyright © 2020 He et al. 2020 He et al. This content is distributed under the terms of the Creative Commons Attribution 4.0 International license.

### Mapping DEGs to metabolic pathways of *N. bombycis*.

**FIG 9:**
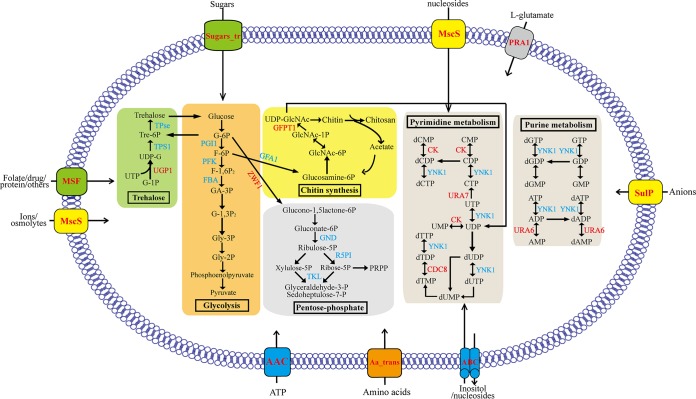
Differentially expressed genes (DEGs) involved in major metabolic pathways during development from the mature spore to the sporoplasm. The DEGs were mapped to the KEGG pathway database. Significantly changed pathways involved trehalose synthesis metabolism (green), glycolysis (orange), pentose phosphate (gray), purine and pyrimidine metabolism (brown), and chitin synthesis (yellow). The metabolites are in black. The upregulated enzymes are highlighted in red, and downregulated proteins are in blue (*P < *0.05).

Interestingly, many downregulated genes were predominantly key enzymes involved in trehalose synthesis metabolism, glycolysis, the pentose phosphate pathway, and chitin synthesis (Table S4 and Fig. 9). The expression levels of 10 transporter genes were upregulated, including energy substance-related transporters such as ADP/ATP carrier protein, mechanical sensitive ion channel proteins, and an amino acid transporter (Table S4 and Fig. 9). This result revealed that sporoplasms may inhibit their own carbon metabolic activity and obtain the substances needed for proliferation through transporter proteins located on the surface of the plasma membrane. Four enzymes were mapped to purine and pyrimidine metabolism, of which three enzymes were upregulated in the sporoplasm. The two pathways are important for nucleotide synthesis, which suggests that the sporoplasm may be preparing for DNA replication.

## DISCUSSION

Microsporidia have been studied for more than 150 years, but relatively little attention has been paid to the sporoplasm. Some reasons for this include the fact that of the more than 1,500 species, the spores of only a small number of these species can be activated *in vitro* using germination solution, and the majority of these solutions activate only a small percentage of spores (15, 31). In addition, sporoplasms are small and lack chitin, which leads to difficulties in their isolation and observation. In the present study, we established a simple method to isolate and observe sporoplasms based on the capability of *N. bombycis* spores to germinate *in vitro*. Spore germination is crucial for the invasion and proliferation of microsporidia. The unique germination process of microsporidia leads to the sporoplasm undergoing a process of compression. The present results showed that the sporoplasm is a flexible stage and the nucleus is compacted after spore germination, which is advantageous for extrusion through the polar tube and in preparation for subsequent proliferation (32). The meront of *N. bombycis* is in direct contact with the host cytoplasm (23). Glycoproteins play an important role in the process of adhesion and recognition, but the present results indicate that the sporoplasm lacks lectin-binding sites, which we speculate may help the sporoplasm evade host immune recognition.

Microsporidia are obligate intracellular parasites, and their genomes have become highly reduced with regard to protein-coding genes as a result (6–8, 33, 34). Recent analyses suggest that most gene loss occurred in the common ancestor of microsporidia, leaving genes for glycolysis in some species (8). Subsequent experimental analysis suggests that core-carbon metabolism predominantly in the spores is not used for ATP synthesis in actively replicating parasites (29, 30). The important human pathogen Enterocytozoon bieneusi has lost glycolysis altogether and thus has no independent means of synthesizing its own ATP (9). The loss of indigenous pathways for energy generation implies that intracellular parasites must obtain those substrates from the infected host cell (33). To adapt to intracellular parasitic life, microsporidia may utilize the hyperpermeability of the plasma membrane to absorb nutrients directly from the cell, and infective sporoplasms would regulate their own metabolic activity and the expression of transporter genes to support parasite growth and replication (7). Genome analyses suggest that expansion of transporter gene families, such as nucleotide transport (NTT) proteins, can compensate for a reduction in metabolic capabilities (7, 27, 28, 35). The freeze-fracture technique has revealed that the plasma membrane of microsporidia contains intramembranous particles which are extremely numerous in immature spores but are almost absent in mature spores (36). The intramembranous particles are interpreted to be transport proteins, which can be expected to decrease during spore maturation (36). Transcriptome analysis of ungerminated and germinated spores of *N. bombycis* demonstrated that protein dephosphorylation is associated with spore germination in microsporidia (25). Using Western blotting, we also detected protein posttranslational modification, ubiquitination, and phosphorylation in mature spores and sporoplasm, which suggested that the ubiquitination level of the total spore proteins increased, while the phosphorylation level of total spore proteins decreased in the sporoplasm. Thus, sporoplasms have evolved and actively regulate a series of reactions to adapt to intracellular parasitic life. Because sporoplasms are obtained *in vitro*, our results from the transcriptome analysis may explain the difference compared to the sporoplasm *in vivo*. When the sporoplasm enter the host cell, changes in sporoplasm gene expression may be caused by environmental stress or host immune response. However, we think that the major gene expression features of *in vivo* sporoplasm can surveyed from the *in vivo* sporoplasm. Then, DEG-related genes involved in glycolysis and transporters were verified using RT-qPCR at 6 h postinfection in infected host cells, which are consistent with the DEGs analyzing *in vitro* sporoplasm (Fig. S6).

10.1128/mSphere.00958-19.6 FIG S6 Relative expression levels of 8 selected genes involved in glycolysis and transporter measured by RT-qPCR at 6 h postinfection. Statistically significant differences are indicated with asterisks (*, *P* < 0.05; **, *P* < 0.01). Download FIG S6, TIF file, 0.1 MB. Copyright © 2020 He et al. 2020 He et al. This content is distributed under the terms of the Creative Commons Attribution 4.0 International license.

Microsporidia may invade host cells by a variety of means. The main hypotheses include direct penetration of the host cell through the polar tube, polar tube interaction with the host cell plasma membrane and sporoplasm endocytosis, and spore adherence to the host cell and invasion by endocytosis (12, 37, 38). However, the molecular mechanism of these invasion methods is unknown, and the role that the sporoplasm plays in the infection process has not been widely reported. Gaining entry to the host cell by endocytosis and escaping from the phagocytic vacuole to infect the cytoplasm have been observed in Encephalitozoon cuniculi by double-immunofluorescence staining (10). *N. bombycis* can gain entry to the host cell by phagocytosis, and the phagocytic uptake of spores might represent a defense mechanism of the host cell (37). In Anncaliia algerae, the process of phagocytosis of the sporoplasm into the host cell by pseudopods has been observed, which is consistent with our findings (15, 17). However, our results cannot prove whether this is a microsporidium-mediated event or a host cell-mediated immune-like response. More experimental evidence is needed to understand the process of phagocytosis of sporoplasm.

In summary, the present study represents the first successful isolation of sporoplasm in microsporidia and will contribute to a comprehensive and in-depth understanding of sporoplasm at the morphological and molecular levels. Our study provides important information for understanding the biology and pathogenesis of microsporidia.

## MATERIALS AND METHODS

### Preparation and purification of Nosema bombycis.

Mature spores of *N. bombycis* isolate CQ1, obtained from infected silkworms in Chongqing, China, were conserved in the China Veterinary Culture Collection Center (CVCC no. 102059), and the harvested spores were purified on a discontinuous Percoll gradient (30%, 45%, 60%, 75%, and 90% [vol/vol]) and centrifuged at 16,000 × *g* for 30 min. Then, spores were collected and washed at least thrice with ultrapure water to remove Percoll. The purified spores were stored at 4°C until further use.

### Purification of sporoplasm.

First, 1 × 10^9^ spores were stained with Hoechst 33258 (Beyotime Biotechnology, China) for 20 min at room temperature in the dark and washed with phosphate-buffered saline (PBS) twice. The spores were suspended in 0.1 M KOH and incubated at room temperature for 40 min to induce extrusion of the sporoplasm. The suspension was centrifuged at 3,000 rpm for 5 min, the supernatant was discarded, and four volumes of cell medium (TC100; USBiological) was added to the tubes. Following incubation, the mixture (sporoplasm, empty spore shells, and nongerminated spores) was observed and photographed using an Olympus FV1200 laser scanning confocal microscope. The ImageJ software was used to measure the diameter of the sporoplasms.

To purify sporoplasms, the spore suspension was added to a discontinuous Percoll gradient and centrifuged at 13,000 × *g* for 20 min. Each gradient region was collected with a pipettor and then diluted in cell medium for centrifugation at 3,000 rpm for 5 min. The sporoplasm suspension was observed and photographed using a confocal microscope.

### Fluorescent staining of spores and sporoplasm.

To analyze the features of the plasma of sporoplasms, different cell dyes were used to stain the sporoplasm. Mature spores and sporoplasms were stained with Hoechst 33258 diluted 1:10,000, propidium iodide (PI) diluted 1:1,000 (red fluorescence), wheat germ agglutinin (WGA) conjugated to Alexa Fluor 488 diluted 1:1,000, and DiI diluted 1:1,000 for 20 min at room temperature in the dark. After washing twice with PBS, a confocal microscope was used to observe and photograph the stained spores and sporoplasm.

### SEM and TEM.

After removal of the supernatant (TC100 cell medium), the pellets containing spore and sporoplasm were then fixed in 2.5% glutaraldehyde for 4 h at room temperature and washed with 0.1 M PBS buffer (pH 7.4) four times (15 min each). For scanning electron microscopy, the pellets were fixed in 1% osmium tetroxide for 1 h. The samples were dehydrated using a graded series of ethanol (30%, 40%, 50%, 60%, 70%, 80%, and 90%) for 10 min each and 100% ethanol two times for 15 min each. Following, the samples were dehydrated using a graded series of tert-butyl alcohol (50%, 75%, and 100%) and tert-butyl alcohol:acetonitrile (2:1 and 1:1), followed by absolute acetonitrile for 10 min each. The dried specimens were coated with gold and transferred to SEM stubs. SEM investigations were carried out using an S-3000N microscope. For transmission electron microscopy, the pellets were fixed in 1% osmium tetroxide for 2 h and washed with 0.1 M PBS buffer. Then, the samples were dehydrated using an ascending ethanol series and 100% acetone two times. They were infiltrated with gradient Epon812 resin (SPI, USA), sequentially embedded in absolute resin, and polymerized at 70°C for 48 h. Ultrathin sections were cut using a Leica EM UC7 ultramicrotome and placed on nickel grids. They were then stained in 3% uranyl acetate, followed by lead citrate. The stained grids were rinsed six times in double-distilled water (ddH_2_O), dried, examined, and then photographed with a JEM-1400 Plus transmission electron microscope at an accelerating voltage of 80 kV. SEM and TEM was performed as previously described (39).

### RNA preparation for transcriptome sequencing.

Total RNA was extracted from each sample using the standard TRIzol extraction protocol, with the addition of a bead-beating step. The integrity and concentration of all RNA samples were assessed by gel electrophoresis and analyzed using a 2100 Bioanalyzer RNA Nanochip. Poly(A) RNA was isolated from 3 μg of purified total RNA. Libraries were prepared using the NEBNext Ultra RNA library prep kit for Illumina (NEB, Ipswich, MA, USA) and sequenced on an Illumina HiSeq 2000 platform, generating 125-bp/150-bp paired-end reads. Three independent library constructions were performed for the MS and SP groups, respectively. Raw data in the fastq format were first processed using in-house perl scripts. In this step, clean data were obtained by the removal of reads containing the adapter, reads containing poly(N), and low-quality reads from the raw data.

### Differential expression analysis.

Determining the expected number of fragments per kilobase per million (FPKM) is currently the most commonly used method for the estimation of gene expression levels. Differential expression analysis of genes between the MS and SP groups was performed using the DESeq2 R package (40). The resulting *P* values were adjusted using the Benjamini-Hochberg approach for controlling the false-discovery rate. Genes with an adjusted *P* value of <0.05 detected by DESeq2 were considered to be differentially expressed.

### GO and KEGG enrichment analysis of differentially expressed genes.

Gene Ontology (GO) enrichment analysis of differentially expressed genes was implemented by the clusterProfiler R package, in which gene length bias was corrected. GO terms with a corrected *P* value of less than 0.05 were considered significantly enriched by differential expressed genes. We used the clusterProfiler R package to test the statistical enrichment of differential expression genes in KEGG pathways.

### Real-time quantitative PCR analysis.

In order to validate the RNA-seq data, 11 randomly selected candidate DEGs were performed to RT-qPCR analysis. Eleven primers were designed and are listed in Table S1. The total RNA of mature spores and sporoplasms was extracted according to the manufacturer’s instructions described above. The cDNA was synthesized with 1 μg total RNA using the GoScript reverse transcription system kit (Promega) after DNA digestion with DNase I. The *N. bombycis SSU* (*NbSSU*) gene was used as a reference in the RT-qPCR experiments. Transcription levels were calculated by the 2^−ΔΔ^*^CT^* value method using three replicates. All statistical *t* tests were performed with GraphPad Prism version 6.0 by two-tailed comparison tests, and any diﬀerence with a *P* value of <0.05 was considered significant (35). The expression patterns of all the selected genes measured by RNA-seq and RT-qPCR were similar, and the results are consistent with the DEG analysis results.

10.1128/mSphere.00958-19.7 TABLE S1 PCR primers used for RT-qPCR validation. Download Table S1, DOCX file, 0.1 MB. Copyright © 2020 He et al. 2020 He et al. This content is distributed under the terms of the Creative Commons Attribution 4.0 International license.

10.1128/mSphere.00958-19.8 TABLE S2 All genes detected by RNA sequencing of the mature spore and sporoplasm transcriptomes. Download Table S2, XLSX file, 0.9 MB. Copyright © 2020 He et al. 2020 He et al. This content is distributed under the terms of the Creative Commons Attribution 4.0 International license.

10.1128/mSphere.00958-19.9 TABLE S3 All differentially expressed genes detected between the mature spore and sporoplasm transcriptomes. Download Table S3, XLSX file, 0.1 MB. Copyright © 2020 He et al. 2020 He et al. This content is distributed under the terms of the Creative Commons Attribution 4.0 International license.

10.1128/mSphere.00958-19.10 TABLE S4 Functional categorization of differentially expressed genes by GO function enrichment and KEGG pathway enrichment. Differentially expressed genes involved in major metabolic pathways during development from the mature spore to the sporoplasm are shown. Download Table S4, XLSX file, 0.1 MB. Copyright © 2020 He et al. 2020 He et al. This content is distributed under the terms of the Creative Commons Attribution 4.0 International license.

### Data availability.

The raw data generated in this study have been submitted to the NCBI Sequence Read Archive under the accession number PRJNA591497.
