# Supplementary figures and images for: Morphology and Transcriptome Analysis of Nosema bombycis Sporoplasm and Insights into the Initial Infection of Microsporidia
Source: mSphere. 2020 Feb 12;5(1):e00958-19. doi: 10.1128/mSphere.00958-19 (PMC7021473; doi:10.1128/mSphere.00958-19)

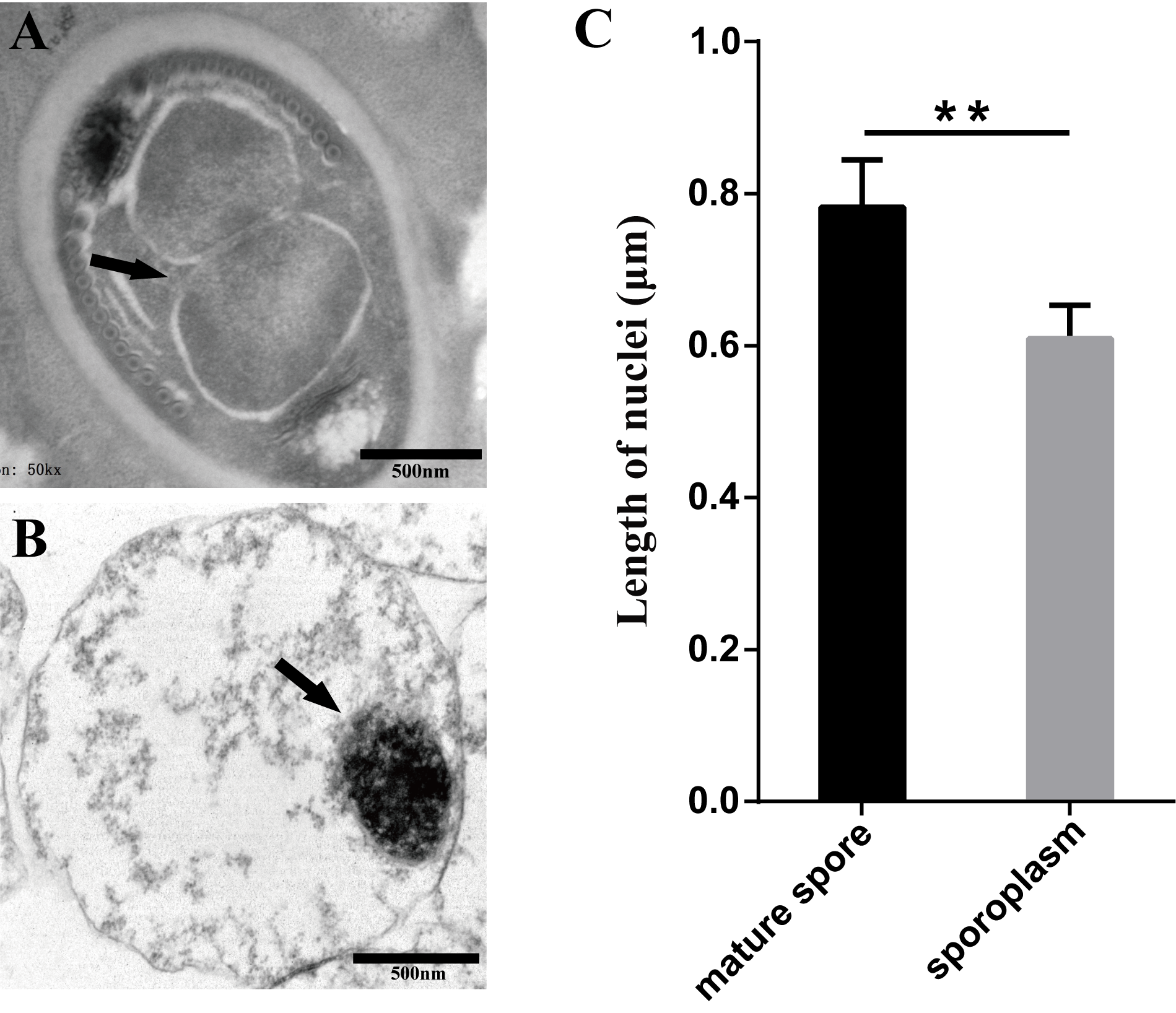

Supplement: FIG S1 [file mSphere.00958-19-sf001.tif]

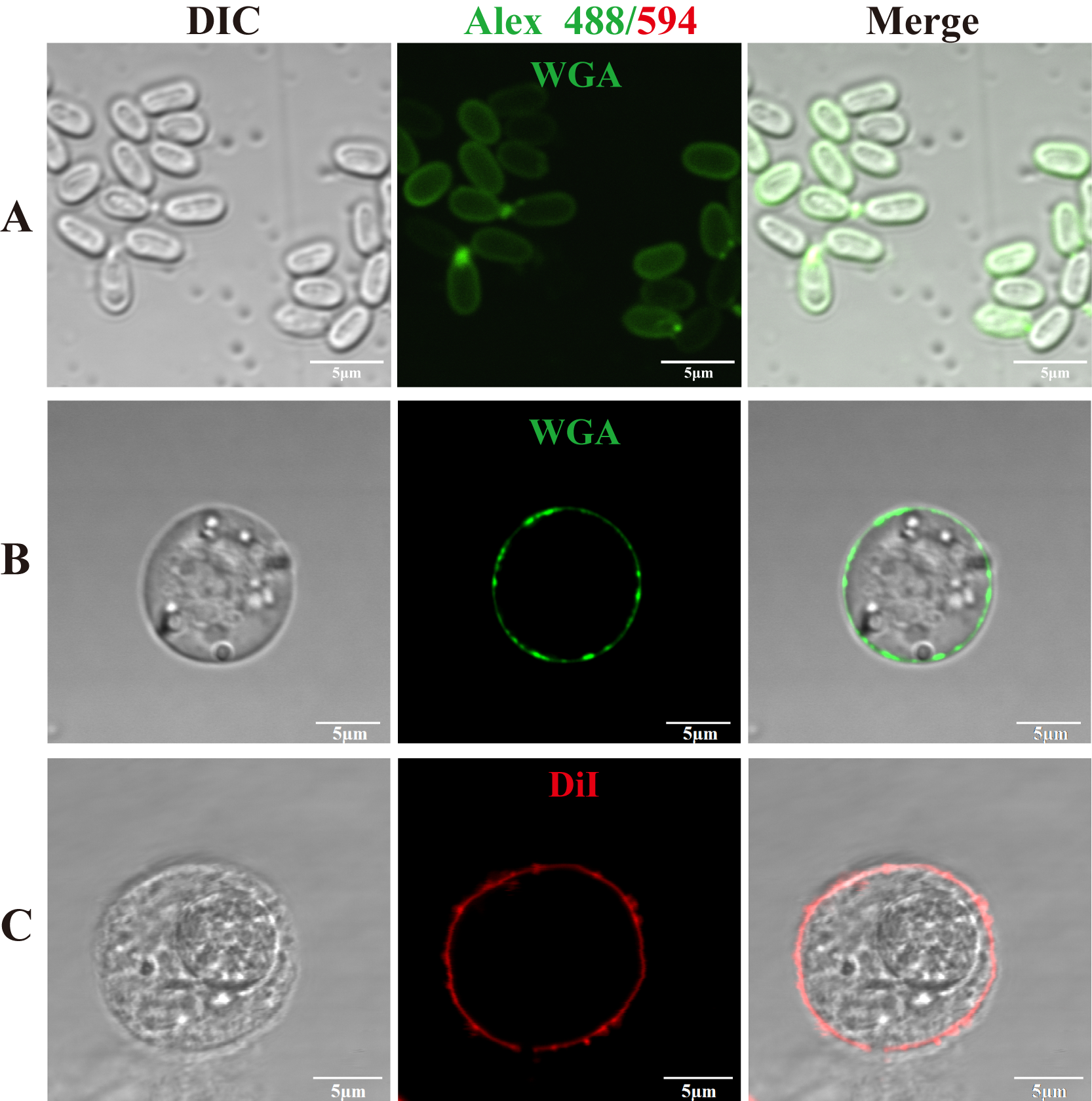

Supplement: FIG S2 [file mSphere.00958-19-sf002.tif]

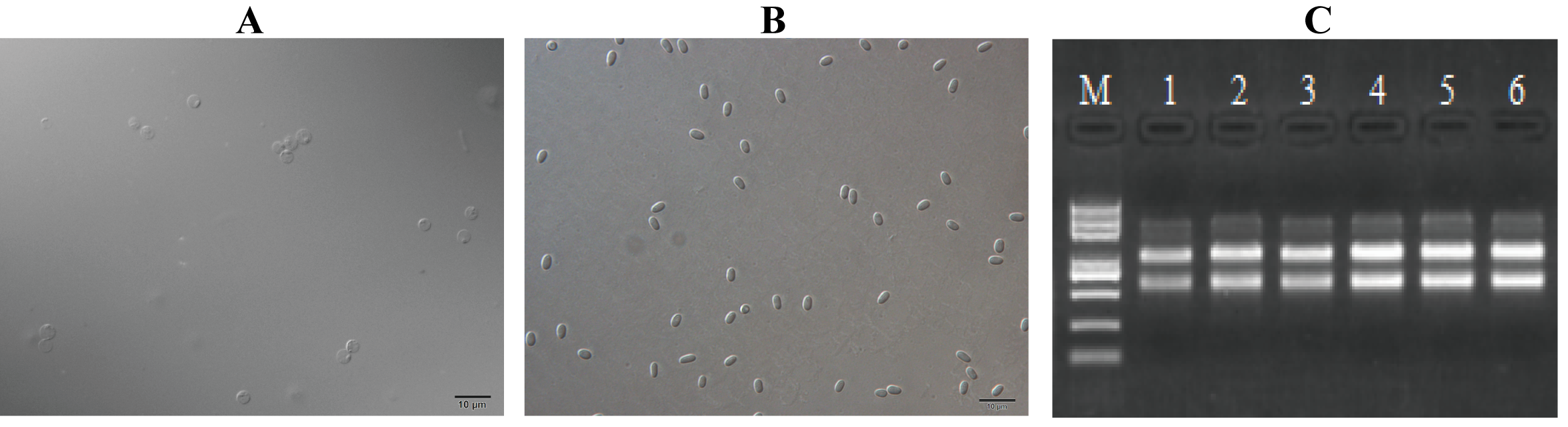

Supplement: FIG S3 [file mSphere.00958-19-sf003.tif]

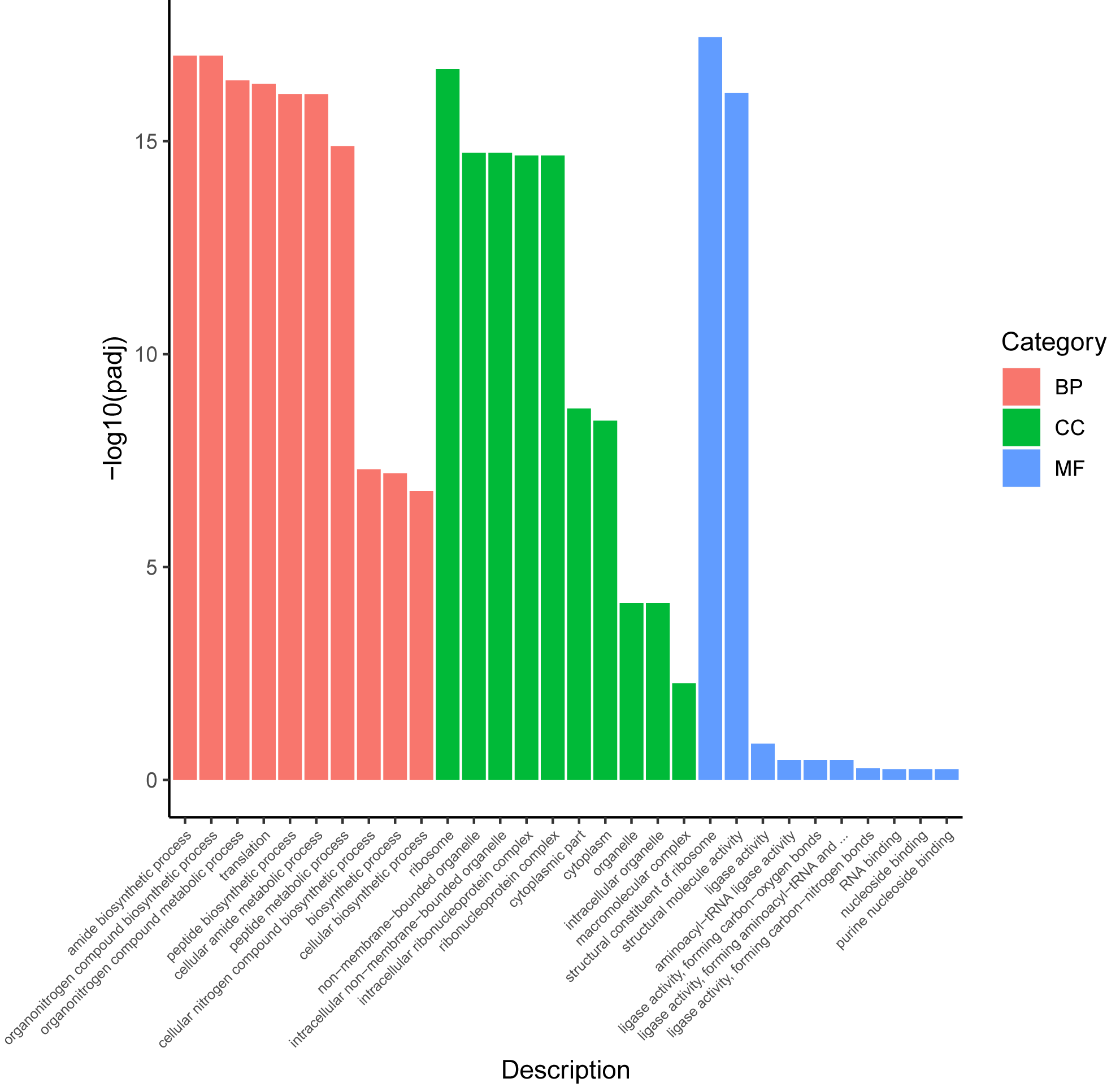

Supplement: FIG S4 [file mSphere.00958-19-sf004.tif]

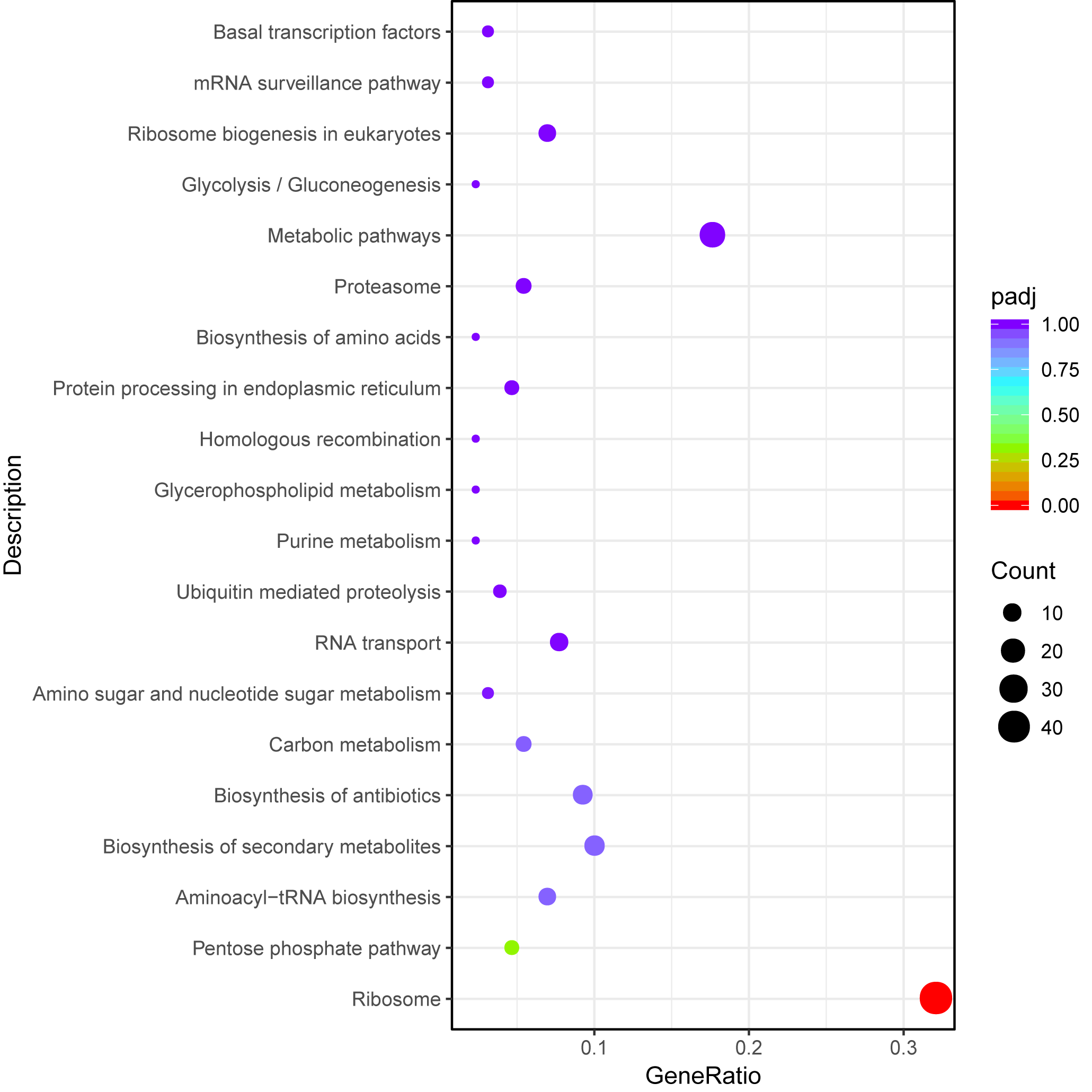

Supplement: FIG S5 [file mSphere.00958-19-sf005.tif]
