# Supplementary material for: Morphology and Transcriptome Analysis of Nosema bombycis Sporoplasm and Insights into the Initial Infection of Microsporidia
Source: mSphere. 2020 Feb 12;5(1):e00958-19. doi: 10.1128/mSphere.00958-19 (PMC7021473; doi:10.1128/mSphere.00958-19)
Supplement: TABLE S1 [file mSphere.00958-19-st001.docx]

Supplementary Table S1. PCR primers used for RT-qPCR validation

| Genes | Primer sequences |
| --- | --- |
| ATP-binding cassette sub-family G member 1 | F-TATACAACGGCAAAGAGCG |
|  | R-GCAGAGTGTGAAT CAAGTCCA |
| Spore wall protein 9 | F-TTCCCTACTGTCATTCATTCTACTG |
|  | R-TCATTCCATCGGTTTTCTCG |
| NBO_7g0021 | F-GGTGTTGTTTTATTTTCAGG |
|  | R-TACTTTGTTTTTTCTTCGTG |
| NBO_555g0004 | F-AGAAGTTTTTGGTGGAGA |
|  | R-ATGACATAATAGATCGGG |
| amino acid permease | F-GTTTTCAGTTTCACTTGCCATCA |
|  | R-TGGTCTTCTGGTTGTGCTTCTA |
| ADP/ATP carrier protein 1 | F-CAACCGACAGTCAGGGGAGA |
|  | R-ATGATGGAAAACATACCAGCAA |
| ATP-binding cassette sub-family B member 7, mitochondrial | F-AAAAGGCTGAGTATTGCTGTGG |
|  | R-TTTCCCCATTTCTGTTAGTCGTT |
| NBO_80g0018 | F-CTCCCCCTTATAACAATGCTCC |
|  | R- AAAAGGGGCCAAAAGACGT |
| Septin 1 | F-GATACACCAGGATTCGGA |
|  | R-CTCTTGTATCTTCAAAAGTAGGA |
| glucose-6-phosphate isomerase | F-CACATCTCTCGGAAGTTGGC |
|  | R-TCTTCTGGAGCCTTCCCTGT |
| trehalose-phosphatase | F-AAGCCGATTGAAACACTCATACA |
|  | R-TTGATTCCATTCGCCCTTG |
| NbSSU | F-CTGGGGATAGTATGATCGCAAGA |
|  | R-CACAGCATCCATTGGAAACG |
